# Design of piecewise deformed elliptical gear with closed pitch curve and its conjugate pair

**DOI:** 10.1038/s41598-022-22139-7

**Published:** 2022-10-13

**Authors:** Youyu Liu, Songsong Lu, Jiao Xu, Siyang Yang, Wanbao Tao

**Affiliations:** 1 Key Laboratory of Advanced Perception and Intelligent Control of High-End Equipment, Ministry of Education, Wuhu, 241000 China; 2 System Office, Wuhu Changxin Technology Co., Ltd, Wuhu, 241000 China; 3 School of Mechanical Engineering, Anhui Polytechnic University, Wuhu, 241000 China

**Keywords:** Engineering, Physics

## Abstract

The family of elliptical gears with closed pitch curves includes elliptical gears and deformed elliptical gears. Elliptical gears are not only the most widely used non-circular gears but also the research hotspot in the field of non-uniform transmission. However, adjusting the shape of its pitch curve is difficult, which seriously restricts its popularization. This article proposes a piecewise deformed elliptical gear with a closed pitch curve, which realizes the unity of all kinds of elliptical gears in the mathematical model, makes the shape of the pitch curve and gear ratio easier to adjust, and expands the connotation and application scope of the family of elliptical gears. Moreover, the design approach of transmission with piecewise deformed elliptical gear was studied, and the inspection method of transmission performance was provided. The internal relationship between piecewise deformed elliptical gears and the family of elliptical gears was then analyzed. Finally, the method of computer-aided design (CAD) system developed was also provided. Additionally, a case was provided to verify the above theories. The designed conjugate pair can realize a correct meshing and adjust the gear ratio more flexibly, laying a theoretical foundation to expand the application field of non-uniform transmission.

## Introduction

The reference surface of a non-circular gear is not a rotating surface^1^. The conjugate pair can realize time-varying meshing transmission based on the established law. It is one of the approaches to realize transmission with a non-uniform ratio. There are many kinds and complex shapes of pitch curves of non-circular gears, which mainly include free pitch curves and typical-shape ones^2^. The pitch curves of the former have no explicit function^3,4^ and are limited by the correct meshing conditions of the conjugate pair, so they are difficult to design and manufacture. The pitch curves of the latter (e.g., elliptical gear) can be expressed by functions^5^, which has numerous applications. Among them, the family of elliptical gears is widely used due to their excellent performances.

Elliptical gears are the most basic and widely used non-circular gears in the elliptic family. Through long-term and in-depth research, scholars have established mature transmission theory and developed many excellent mechanisms with planetary Elliptical gears. Liu et al. have analyzed the dynamic characteristics of elliptical gears, and the general factors including torque, rotation speed and tooth root stress^6^. However, their gear ratio should not be too large, and the gear ratio can only circulate for one cycle when the driving gear rotates for one cycle^7^, limiting the scope of the application. At present, most scholars are committed to improving their transmission performance.

To meet the needs of different transmission conditions, high-order elliptical gears were developed based on elliptical gears. When the driving gear rotates for one cycle, the driven gear will have multiple symmetrical rotation cycles. Different order and eccentricity of them will change the kinematic parameters and transmission characteristics of high-order elliptical gears, which can be used in gear pumps to reduce the flow ripples^8^.

Unlike elliptical gears, the pitch curve of a deformed elliptical gear is a continuous closed curve that is composed of two ellipses with different orders^9^, and the gear ratio is also a two-segment asymmetric curve. This unique feature provides convenience for mechanical creative design. For example, Feng et al. adjusted the relevant parameters such as the semimajor axis, eccentricity, and modified coefficient of a deformed elliptical gear, thus optimizing the dynamic performance of the pricking hole with a liquid fertilizer applicator for deep-into types^10^. The adjusting ability of the gear ratio of a deformed elliptical gear is much weaker than that of a non-circular gear with a free pitch curve^11^. However, the shapes of non-circular gears with a free pitch curve are different^2^, bringing insurmountable difficulties to the standardized design and manufacture.

Based on the high-order elliptical gears and deformed elliptical gears, Zhang et al. have proposed the concept and design method of high-order deformed elliptical gears, and realized the periodic transmission of deformed elliptical gears^12^. The pitch curve in each cycle is deformed into two segments, but its shape is still difficult to change freely.

Given that the pitch curve of the deformed elliptical gear is divided into three segments or more, it is expected to further approach the non-circular gear with a free pitch curve to overcome their respective shortcomings while retaining their advantages. This article extends the elliptical gear with a closed pitch curve and proposes a piecewise deformed elliptical gear. The unification of all kinds of elliptical gear families on the mathematical model will be realized.

The equation of a pitch curve of the high-order elliptical gear in the transverse plane is as follows^13^:

1 $$r=\frac{p}{1-e\,\mathrm{cos}\left(n\theta \right)}$$

where, $$p=A\left(1-{e}^{2}\right)$$.

$N$ (positive integer) ellipses with different orders ($n$, not necessarily an integer) are formed into a continuous closed curve and then form an $N$-segment deformed ellipse ($N\ge$ 3) called a piecewise deformed ellipse. It is expressed in Eq. (2), which is extended from Eq. (1).

2 $$\left\{\begin{array}{l}{r}_{1}=\frac{p}{1-e\,\mathrm{cos}\,{\Psi }_{1}}\quad  \theta \in \left(0\right.,\left.\frac{2\pi }{N{m}_{1}}\right]\\ {r}_{j}=\frac{p}{1-e\,\mathrm{cos}\,{\Psi }_{j}} \quad  \theta \in \left({\sum }_{k=1}^{j-1}\frac{2\pi }{N{m}_{k}}\right.,\left.{\sum }_{k=1}^{j}\frac{2\pi }{N{m}_{k}}\right]\end{array}\right.$$

where, $$j=\mathrm{2,3}\dots N,{\Psi }_{1}={m}_{1}\theta $$, $${\Psi }_{j}={m}_{j}\left(\theta -\sum_{k=1}^{j-1}\frac{2\pi }{N{m}_{k}}\right)+\frac{2\left(j-1\right)\pi }{N}$$.

${m}_{j}$ in Eq. (2) must satisfy Eq. (3), and ${m}_{j}>1/N$.

3 $${\sum }_{j=1}^{N}\frac{1}{{m}_{j}}=N$$

According to Eq. (2), when $\theta ={\sum }_{k=1}^{j-1}\frac{2\pi }{N{m}_{k}}$,

4 $${r}_{j}={r}_{j-1}=\frac{p}{1-e\,\mathrm{cos}\,\left[2\left(j-1\right)\pi /N\right]}$$

while $$\theta =0$$ and $$2\pi $$,

5 $${r}_{1}={r}_{N}=A\left(1+e\right)$$

It can be seen that each segment of modified ellipses is connected continuously when $\theta ={\sum }_{k=1}^{j-1}\frac{2\pi }{N{m}_{k}}$ and $\theta =0\left(2\pi \right)$ and then forms an end-to-end closed curve.

**Figure 1:**
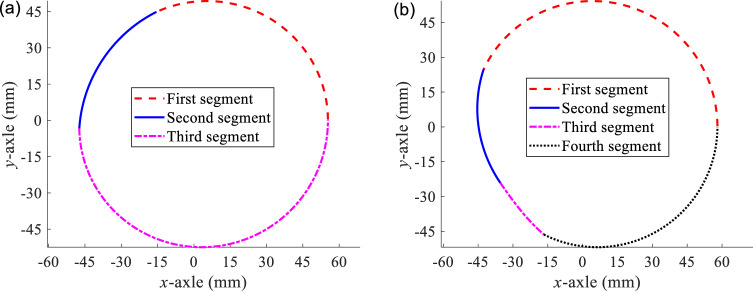
Pitch curves of piecewise deformed elliptical gears, (**a**) $\mathrm{A}$= 50, $\mathrm{e}$= 0.1, ${\mathrm{m}}_{1}$= 1.1, ${\mathrm{m}}_{2}$= 1.6, ${\mathrm{m}}_{3}$= 0.682, and (**b**) $\mathrm{A}$= 50, $\mathrm{e}$ = 0.15, ${\mathrm{m}}_{1}$= 0.6, ${\mathrm{m}}_{2}$ = 1.4, ${\mathrm{m}}_{3}$= 2.5, ${\mathrm{m}}_{4}$ = 0.820.

By adjusting $A$, $e$, $N$, and ${m}_{k}$, an aperiodic non-circular curve can be approached with some piecewise deformed elliptical curves. Figure 1 shows two cases of pitch curves of piecewise deformed elliptical gears, which all conform to the above closed and continuous characteristics. Compared with the deformed elliptical gear ($N$ = 2), the shape of the pitch curve of the piecewise deformed elliptical gear is more flexible, making it more convenient to adjust the gear ratio and meet a variety of transmission requirements.

## Results

### Design of conjugate pair of piecewise deformed elliptical gear

Equation (6) is used to calculate the pitch curve perimeter of a piecewise deformed elliptical gear^14^.

6 $$L={\int }_{0}^{2\pi }\sqrt{{r}^{2}+{\left(\frac{dr}{d\theta }\right)}^{2}}\mathrm{d}\theta $$

After determining the parameters of the driving gear, such as normal modulus (${m}_{n}$), teeth number ($z$), helix angle ($\beta$), and $e$, $N$, $n$, ${m}_{j}$, the pitch curve perimeter must meet Eq. (7) to make the teeth evenly distributed on the gear^2^.

7 $$L=\frac{{m}_{n}\pi z}{\,\mathrm{cos}\,\beta }$$

According to Eqs. (2), (6), and (7) and using the integral function of quadl (L, 0, π/2, tol) in MATLAB, the semimajor axis $A$ of the driving gear can be determined.

The orders of the driving gear and the driven one that constitute the non-circular conjugate pair are 1, namely, $n$ = ${n}^{{\prime}}$= 1. The gear ratio of the conjugate pair is as follows:

8 $$i=\left\{\begin{array}{l}\frac{a\pm p-ae\,\mathrm{cos}\,{\Psi }_{1}}{p}\quad  \theta \in \left(0\right.,\left.\frac{2\pi }{N{m}_{1}}\right]\\ \frac{a\pm p-ae\,\mathrm{cos}\,{\Psi }_{j}}{p}\quad  \theta \in \left({\sum }_{k=1}^{j-1}\frac{2\pi }{N{m}_{k}}\right.,\left.{\sum }_{k=1}^{j}\frac{2\pi }{N{m}_{k}}\right]\end{array}\right.$$

where the “$\pm$” is taken as “−” for external meshing transmission and “−” for internal meshing transmission.

According to the principle of non-circular gear transmission^2^, the pitch curve equation of the driven gear is as follows:

9 $$\left\{\begin{array}{l}{r}^{{\prime}}=a\pm r\\ {\theta }^{{\prime}}={\int }_{0}^{\theta }\frac{1}{i}\mathrm{d}\theta \end{array}\right.$$

The closing condition of the driven gear is as follows:

10 $$2\pi ={\int }_{0}^{2\pi }\frac{1}{i}\mathrm{d}\theta ={\int }_{0}^{\frac{2\pi }{N{m}_{1}}}\frac{p}{a\pm p-ae\,\mathrm{cos}\,{\Psi }_{1}}\mathrm{d}\theta +{\sum }_{j=2}^{N}{\int }_{\sum_{k=1}^{j-1}\frac{2\pi }{N{m}_{k}}}^{\sum_{k=1}^{j}\frac{2\pi }{N{m}_{k}}}\frac{p}{a\pm p-ae\,\mathrm{cos}\,{\Psi }_{j}}\mathrm{d}\theta $$

According to Eq. (10), the center distance ($a$) can be determined. Specifying the interval of $a$ and allowable error ($\delta$), the integral function trapz (function)^15^ in MATLAB is used to integrate Eq. (10). Moreover, $a$ is determined within $\delta$ by using golden section method.

According to Eqs. (9) and (10),

11 $${\theta }^{{\prime}}=\left\{\begin{array}{l}{\int }_{0}^{\theta }\frac{p}{a\pm p-ae\,\mathrm{cos}\,{\Psi }_{1}}\mathrm{d}\theta \quad  \theta \in \left(0\right.,\left.\frac{2\pi }{N{m}_{1}}\right]\\ {\int }_{0}^{\frac{2\pi }{N{m}_{1}}}\frac{p}{a\pm p-ae\,\mathrm{cos}\,{\Psi }_{1}}\mathrm{d}\theta +{\sum }_{j=2}^{N-1}{\int }_{\sum_{k=1}^{j-1}\frac{2\pi }{N{m}_{k}}}^{\sum_{k=1}^{j}\frac{2\pi }{N{m}_{k}}}\frac{p}{a\pm P-ae\,\mathrm{cos}\,{\Psi }_{j}}\mathrm{d}\theta +{\int }_{\sum_{k=1}^{N-1}\frac{2\pi }{N{m}_{k}}}^{\theta }\frac{p}{a\pm p-ae\,\mathrm{cos}\,{\Psi }_{j}}\mathrm{d}\theta \quad \theta \in \left({\sum }_{k=1}^{j-1}\frac{2\pi }{N{m}_{k}}\right.,\left.{\sum }_{k=1}^{j}\frac{2\pi }{N{m}_{k}}\right]\end{array}\right.$$

According to Eqs. (2) and (10),

12 $$\left\{\begin{array}{l}{r}_{1}^{{\prime}}=a\pm \frac{p}{1-e\,\mathrm{cos}\,{\Psi }_{1}}\quad  \theta \in \left(0\right.,\left.\frac{2\pi }{N{m}_{1}}\right]\\ {r}_{j}^{{\prime}}=a\pm \frac{p}{1-e\,\mathrm{cos}\,{\Psi }_{j}} \quad  \theta \in \left({\sum }_{k=1}^{j-1}\frac{2\pi }{N{m}_{k}}\right.,\left.{\sum }_{k=1}^{j}\frac{2\pi }{N{m}_{k}}\right]\end{array}\right.$$

The angular acceleration of the driven gear of a piecewise deformed elliptical gear pair is as follows:

13 $${\alpha }^{{\prime}}=-\frac{{\omega }^{2}}{{i}^{2}}\cdot \frac{\mathrm{d}i}{\mathrm{d}\theta }=\left\{\begin{array}{l}-\frac{p{\omega }^{2}ae{m}_{1}\,\mathrm{sin}{\Psi }_{1}}{{\left(a\pm p-ae\,\mathrm{cos}\,{\Psi }_{1}\right)}^{2}} \quad \theta \in \left(0\right.,\left.\frac{2\pi }{N{m}_{1}}\right]\\ -\frac{p{\omega }^{2}ae{m}_{j}\,\mathrm{sin}{\Psi }_{j}}{{\left\{a\pm p-ae\,\mathrm{cos}\,{\Psi }_{j}\right\}}^{2}} \quad \theta \in \left({\sum }_{k=1}^{j-1}\frac{2\pi }{N{m}_{k}}\right.,\left.{\sum }_{k=1}^{j}\frac{2\pi }{N{m}_{k}}\right]\end{array}\right.$$

According to Eqs. (11) and (12), the number ${N}^{{\prime}}$ of segments in each cycle of the driven gear is equal to $N$. In general, each segment of the pitch curve of a non-circular gear is not periodic.

Therefore, the piecewise deformed elliptical gear can only be used for external meshing transmission, and the above situation of internal meshing is of no practical significance.

### Curvature radius and inspection of convexity of the pitch curve

The curvature radius^2^ of the driving gear’s pitch curve is as follows:

14 $$\rho =\frac{{\left[{r}^{2}+{\left(\frac{\mathrm{d}r}{\mathrm{d}\theta }\right)}^{2}\right]}^\frac{3}{2}}{{r}^{2}+{2\left(\frac{\mathrm{d}r}{\mathrm{d}\theta }\right)}^{2}-r\frac{{\mathrm{d}}^{2}r}{\mathrm{d}{\theta }^{2}}}$$

According to Eq. (2), the first-order and second-order differentiation for the pitch curve of the first segment is formed using Eq. (15).

15 $$\left\{\begin{array}{l}\frac{{\mathrm{d}r}_{1}}{\mathrm{d}\theta }=\frac{{m}_{1}pe\,\mathrm{sin}{\Psi }_{1}}{{\left(1-e\,\mathrm{cos}\,{\Psi }_{1}\right)}^{2}} \\ \frac{{{\mathrm{d}}^{2}r}_{1}}{\mathrm{d}{\theta }^{2}}=\frac{{m}_{1}^{2}p{e}^{2}{\,\mathrm{sin}}^{2}{\Psi }_{1}+{m}_{1}^{2}p{e}^{2}-{m}_{1}^{2}p{e}^{2}\,\mathrm{cos}\,{\Psi }_{1}}{{\left(1-e\,\mathrm{cos}\,{\Psi }_{1}\right)}^{3}}\end{array}\right.$$

Substituting Eq. (15) into Eq. (14), the curvature radius is shown in Eq. (16).

16 $${\rho }_{1}=p\frac{{\left\{{\left[1-e\,\mathrm{cos}\,{\Psi }_{1}\right]}^{2}+{e}^{2}{m}_{1}^{2}{\,\mathrm{sin}}^{2}{\Psi }_{1}\right\}}^\frac{3}{2}}{{\left[1-e\,\mathrm{cos}\,{\Psi }_{1}\right]}^{3}\left[1+e\left({m}_{1}^{2}-1\right)\,\mathrm{cos}\,{\Psi }_{1}\right]}$$

According to Eq. (15), its numerator is always greater than zero. Assuming that the pitch curve is convex, its denominator should also be greater than zero.

Since $0<e<1$, $-1\le \,\mathrm{cos}\,\left({m}_{1}\theta \right)\le 1$, thus ${\left[1-e\,\mathrm{cos}\,\left({m}_{1}\theta \right)\right]}^{3}>0$, the convex condition of the pitch curve is as follows:

17 $$F=1+e\left({m}_{1}^{2}-1\right)\,\mathrm{cos}\,{\Psi }_{1}\ge 0$$

If $\left({m}_{1}^{2}-1\right)cos\left({m}_{1}\theta \right)\ge 0$, then Eq. (17) is set up; if $\left({1-m}_{1}^{2}\right)\,\mathrm{cos}\,{\Psi }_{1}<0$, then,

18 $$e\le \frac{1}{\left({1-m}_{1}^{2}\right)\,\mathrm{cos}\,{\Psi }_{1}}$$

Therefore, the convexity condition of each segment curve is as follows:

19 $$e\le \mathrm{min}\left\{\frac{1}{\left({1-m}_{j}^{2}\right)\,\mathrm{cos}\,{\Psi }_{j}}\right\} \left({1-m}_{j}^{2}\right)\,\mathrm{cos}\,{\Psi }_{j}>0$$

A non-circular gear meeting Eq. (19) can be processed by hobbing.

According to Eq. (14), the minimum curvature radius of each segment can be obtained, and that of the driving gear is as follows:

20 $${\rho }_{\mathrm{min}}=\mathrm{min}\left\{{{\rho }_{j}}_{\mathrm{min}}\right\}$$

Similarly, the curvature radius of the pitch curve of the driven gear is as follows:

21 $${\rho }^{{\prime}}=\frac{{\left[{{r}^{{\prime}}}^{2}+{\left(\frac{\mathrm{d}{r}^{{\prime}}}{\mathrm{d}{\theta }^{{\prime}}}\right)}^{2}\right]}^\frac{3}{2}}{{{r}^{{\prime}}}^{2}+2{\left(\frac{\mathrm{d}{r}^{{\prime}}}{\mathrm{d}{\theta }^{{\prime}}}\right)}^{2}-{r}^{{\prime}}\frac{{\mathrm{d}}^{2}{r}^{{\prime}}}{\mathrm{d}{{\theta }^{{\prime}}}^{2}}}$$

In consideration of

22 $$\left\{\begin{array}{l}\frac{\mathrm{d}{r}^{{\prime}}}{\mathrm{d}{\theta }^{{\prime}}}=\frac{\mathrm{d}{r}^{{\prime}}}{\mathrm{d}\theta }\cdot \frac{\mathrm{d}\theta }{\mathrm{d}{\theta }^{{\prime}}}=\frac{\mathrm{d}{r}^{{\prime}}}{\mathrm{d}\theta }\cdot i \\ \frac{{\mathrm{d}}^{2}{r}^{{\prime}}}{\mathrm{d}{{\theta }^{{\prime}}}^{2}}=\frac{{\mathrm{d}}^{2}{r}^{{\prime}}}{\mathrm{d}{\theta }^{2}}\cdot {i}^{2}+\frac{\mathrm{d}{r}^{{\prime}}}{\mathrm{d}\theta }\cdot \frac{\mathrm{d}i}{\mathrm{d}\theta }\cdot i\end{array}\right.$$

According to Eq. (12), for the pitch curve in the first segment,

23 $$\left\{\begin{array}{l}\frac{\mathrm{d}{r}_{1}^{{\prime}}}{\mathrm{d}\theta }=\frac{-pe{m}_{1}\,\mathrm{sin}{\Psi }_{1}}{{\left[1-e\,\mathrm{cos}\,{\Psi }_{1}\right]}^{2}} \\ \frac{{\mathrm{d}}^{2}{r}_{1}^{{\prime}}}{\mathrm{d}{\theta }^{2}}=\frac{-pe{m}_{1}^{2}\left[2e{\,\mathrm{sin}}^{2}{\Psi }_{1}-\,\mathrm{cos}\,{\Psi }_{1}\right]\left(1-e\,\mathrm{cos}\,{\Psi }_{1}\right)}{{\left[1-e\,\mathrm{cos}\,{\Psi }_{1}\right]}^{3}}\end{array}\right.$$

By substituting Eq. (23) into Eq. (21), the curvature radius of the pitch curve of the first segment of a driven gear can be obtained. In the same way, the curvature radius of other segments of a pitch curve can also be obtained. In the specific design, the convex condition of the curve can be judged according to the positive and negative symbols of each segment’s curvature radius, and then the correct processing method can be selected.

### Inspection of a pressure angle

A pressure angle ($\gamma$) is an intersection angle between the absolute speed direction of the driven gear and the driving gear at the contact point of their pitch curves and the normal direction of their tooth profiles. According to reference ^16^,

24 $$\gamma =\mu +{\alpha }_{n}-\frac{\pi }{2}$$

where $\mu$ is the diameter-tangent angle^17^ of the driving gear, namely, an angle between the polar radius and the positive direction (in which angle $\theta$ increases) of the tangent of the diving gear’s pitch curve.

According to the geometry of non-circular gear transmission and Eq. (2),

25 $$\mu =\left\{\begin{array}{l}{\,\mathrm{cos}\,}^{-1}\frac{-e{m}_{1}\,\mathrm{sin}{\Psi }_{1}}{\sqrt{{\left(1-e\,\mathrm{cos}\,{\Psi }_{1}\right)}^{2}+{m}_{1}^{2}{e}^{2}{\,\mathrm{sin}}^{2}{\Psi }_{1}}} \quad  \theta \in \left(0\right.,\left.\frac{2\pi }{N{m}_{1}}\right]\\ {\,\mathrm{cos}\,}^{-1}\frac{-e{m}_{j}\,\mathrm{sin}{\Psi }_{j}}{\sqrt{{\left(1-e\,\mathrm{cos}\,{\Psi }_{j}\right)}^{2}+{m}_{j}^{2}{e}^{2}{\,\mathrm{sin}}^{2}{\Psi }_{j}}}  \quad  \theta \in \left({\sum }_{k=1}^{j-1}\frac{2\pi }{N{m}_{k}}\right.,\left.{\sum }_{k=1}^{j}\frac{2\pi }{N{m}_{k}}\right]\end{array}\right.$$

The larger the $\gamma$, the smaller the moment of force, and the greater the force when transmitting the same torque. Consequently, the load acting on the bearing will increase accordingly. When $\gamma$ is too large, it may even produce self-locking, making the conjugate pair unable to drive. To avoid this phenomenon, ${\gamma }_{max} \le$ 65° is usually required^14^, and then $\mu \in$[45°, 135°].

### Inspection of root undercutting

The curvature radius of each point on the pitch curve of a non-circular gear is different, and the most likely root undercutting is the part with the smallest curvature radius. For the driving gear, ${\rho }_{\mathrm{min}}=\mathrm{min}\left\{{\rho }_{j\mathrm{min}}\right\}$. For the driven gear, ${\rho }_{\mathrm{min}}=\mathrm{min}\left\{{\rho }_{j\mathrm{min}}^{{\prime}}\right\}$. Equations (26) and (27) can be used to calculate the non-undercutting condition for gear hobbing with external meshing and full-convex pitch curve^18^.

26 $${m}_{t\mathrm{max}}\le \frac{{\rho }_{\mathrm{min}}{\,\mathrm{sin}}^{2}{\alpha }_{t}}{{h}_{at}^{*}}=\frac{{\rho }_{\mathrm{min}}{\mathrm{tan}}^{2}{\alpha }_{n}}{\left({\,\mathrm{cos}\,}^{2}\beta +{\mathrm{tan}}^{2}{\alpha }_{n}\right){h}_{an}^{*}\,\mathrm{cos}\,\beta }$$

27 $${m}_{n\mathrm{max}}\le \frac{{\rho }_{\mathrm{min}}{\,\mathrm{sin}}^{2}{\alpha }_{n}}{\left({\mathrm{cos}}^{2}\beta +{\mathrm{tan}}^{2}{\alpha }_{n}\right){h}_{an}^{*}}$$

where ${\alpha }_{n}$ is generally 20° and ${h}_{an}^{*}$ is generally 1.

If gear shaping without undercutting is adopted, the minimum teeth number of a gear-shaping cutter should meet the following:

28 $${z}_{0\mathrm{min}}\ge \frac{{m}_{n}^{2}{{h}_{an}^{*}}^{2}\,{\mathrm{cos}}^{3}\, \beta +{m}_{n}^{2}{{h}_{an}^{*}}^{2} \, \mathrm{cos}\, \beta {\mathrm{tan}}^{2}{\alpha }_{n}-{\rho }_{\mathrm{min}}^{2}{\mathrm{tan}}^{2}{\alpha }_{n}\,\mathrm{cos}\beta }{{\rho }_{\mathrm{min}}{\mathrm{tan}}^{2}{\alpha }_{n}{m}_{n}-{m}_{n}^{2}{h}_{an}^{*}{\,\mathrm{cos}}^{2}\beta -{m}_{n}^{2}{h}_{an}^{*}{\mathrm{tan}}^{2}{\alpha }_{n}}$$

### Inspection of contact ratio

The definition and calculation of a meshing contact ratio^2^ of a piecewise deformed elliptical gear are the same as that of an ordinary non-circular gear; see Eq. (29) for its calculation.

The condition for continuous transmission of non-circular gears is as $\varepsilon$ > l^19^. Since $\rho$ and ${\rho }^{{\prime}}$ are different at each point of the pitch curves, and $\varepsilon$ is a function of $\theta$, the contact ratio of each pair of gear teeth should be inspected in the design.

29 $$\varepsilon =\frac{\mu +{\mu }^{{\prime}}}{\pi {m}_{t}\,\mathrm{cos}\,{\alpha }_{t}}=\frac{\left(\mu +{\mu }^{{\prime}}\right)\sqrt{{\mathrm{tan}}^{2}{\alpha }_{n}+{\,\mathrm{cos}}^{2}\,\beta }}{\pi {m}_{n}}$$

See Eq. (30) for $\mu$ and ${\mu }^{{\prime}}$.

30 $$\left.\begin{array}{l}\mu =\sqrt{{\left(\rho +{h}_{a}\right)}^{2}-{\left(\rho \,\mathrm{cos}\,{\alpha }_{t}\right)}^{2}}-\rho \,\mathrm{sin}\,{\alpha }_{t}=\sqrt{{\left(\rho +{h}_{a}\right)}^{2}-\frac{{\rho }^{2}{\,\mathrm{cos}}^{2}v\beta }{{\mathrm{tan}}^{2}{\alpha }_{n}+{\,\mathrm{cos}}^{2}\,\beta }}-\rho \sqrt{\frac{{\mathrm{tan}}^{2}{\alpha }_{n}}{{\mathrm{tan}}^{2}{\alpha }_{n}+{\,\mathrm{cos}}^{2}\,\beta }}\\ {\mu }^{{\prime}}=\sqrt{{\left({\rho }^{{\prime}}+{h}_{a}^{{\prime}}\right)}^{2}-{\left({\rho }^{{\prime}}\,\mathrm{cos}\,{\alpha }_{t}\right)}^{2}}-{\rho }^{{\prime}}\,\mathrm{sin}{\alpha }_{t}=\sqrt{{\left({\rho }^{{\prime}}+{h}_{a}^{{\prime}}\right)}^{2}-\frac{{{\rho }^{{\prime}}}^{2}{\,\mathrm{cos}}^{2}\,\beta }{{\mathrm{tan}}^{2}\,{\alpha }_{n}+ {\,\mathrm{cos}}^{2}\,\beta }}-{\rho }^{{\prime}}\sqrt{\frac{{\mathrm{tan}}^{2}\,{\alpha }_{n}}{{\mathrm{tan}}^{2}{\alpha }_{n}+{\,\mathrm{cos}}^{2}\,\beta }}\end{array}\right\}$$

## Discussion

When $N$=1 and ${m}_{1}$ is a positive integer, Eq. (2) is equivalent to Eq. (1), in which $n$= ${m}_{1}$. It is essentially a pitch curve of a high-order elliptical gear. The gear ratio of the gear obtained from Eq. (8) is shown as Eq. (31). Based on special conditions, the center distance expression obtained by integrating Eq. (10) is shown as Eq. (32). The pitch curve equation of the driven gear can be obtained from Eqs. (11), (12), and (32) (see Eq. (33)). It can be seen that the driven gear is also a high-order elliptical gear. This high-order elliptical gear belongs to a special case of a family of elliptical gears studied in reference^20^, consistent with the results of this article. Furthermore, when $N$=1 and ${m}_{1}$= 1, the high-order elliptical gear evolves into an elliptical gear.

31 $$i=\frac{a-p-ae\,\mathrm{cos}\left({\Psi }_{1}\right)}{p}$$

32 $$a=A\sqrt{{\left({m}_{1}^{{\prime}}/{m}_{1}\right)}^{2}-{e}^{2}\left[{\left({m}_{1}^{{\prime}}/{m}_{1}\right)}^{2}-1\right]}+A$$

33 $$\left\{\begin{array}{l}{r}^{{\prime}}=\frac{{p}^{{\prime}}}{1+{e}^{{\prime}}\,\mathrm{cos}\left({\Psi }_{1}^{{\prime}}\right)} \\ {\theta }^{{\prime}}=\frac{2}{{m}_{1}^{{\prime}}}\, {\mathrm{tan}}^{-1}\left[\sqrt{\frac{a+p+ae}{a+p-ae}}\, \mathrm{tan}\,  \left(\frac{{\Psi }_{1}}{2}\right)\right] \\ {p}^{{\prime}}=\frac{{\left({m}_{1}^{{\prime}}/{m}_{1}\right)}^{2}p}{\sqrt{{\left({m}_{1}^{{\prime}}/{m}_{1}\right)}^{2}-{e}^{2}\left[{\left({m}_{1}^{{\prime}}/{m}_{1}\right)}^{2}-1\right]}} \\ {e}^{{\prime}}=\frac{e}{\sqrt{{\left({m}_{1}^{{\prime}}/{m}_{1}\right)}^{2}-{e}^{2}\left[{\left({m}_{1}^{{\prime}}/{m}_{1}\right)}^{2}-1\right]}}\end{array}\right.$$

When $N$= 2, Eq. (2) is essentially a pitch curve of the deformed elliptical gear. Equation (32) shows the expression of the center distance of the conjugate pair, Eq. (34) shows the gear ratio obtained from Eq. (8), and Eq. (35) is the pitch curve equation of the driven gear obtained from Eqs. (11), (12), and (32). It can be seen that the driven gear is also a deformed elliptical gear. This kind of gear transmission belongs to a special case of a deformed elliptical gear studied in literature^9^, consistent with the results of this article. Moreover, when $N$= 2 and ${m}_{1}$=${m}_{2}$= 1, the deformed elliptical gear evolves into an elliptical gear.

34 $$i=\left\{\begin{array}{l}\frac{1+{e}^{2}-2e\,\mathrm{cos}\, {\Psi }_{1}}{1-{e}^{2}} \quad \theta \in \left(0\right.,\left.\frac{\pi }{{m}_{1}}\right]\\ \frac{1+{e}^{2}-2e\,\mathrm{cos}\, \left[{m}_{2}\left(\theta -\frac{\pi }{{m}_{1}}\right)+\pi \right]}{1-{e}^{2}}\quad  \theta \in \left(\frac{\pi }{{m}_{1}}\right.,\left.2\pi \right]\end{array}\right.$$

35 $$\left\{\begin{array}{l}{r}_{1}^{{\prime}}=\frac{p}{1+e\,\mathrm{cos}\left({m}_{1}{\theta }^{{\prime}}\right)} \quad {\theta }^{{\prime}}\in \left(0\right.,\left.\frac{\pi }{{m}_{1}}\right]\\ {r}_{2}^{{\prime}}=\frac{p}{1+e\,\mathrm{cos}\left[{m}_{2}\left({\theta }^{{\prime}}-2\pi \right)\right]}\quad {\theta }^{{\prime}}\in \left(\frac{\pi }{{m}_{1}}\right.,\left.2\pi \right]\end{array}\right.$$

When $N$ ≥ 3, the pitch curve of the driven gear is an $N$-segment non-circular gear, which is no longer a piecewise deformed elliptical gear.

Therefore, the segmented deformed elliptical gear and its conjugate pair proposed in this article cover elliptical gear transmission and deformed elliptical gear transmission and expand the number of deformed segments of the pitch curve to more than 3, providing a theoretical basis for the design of a non-circular conjugate pair that meets the requirements of accurate transmission.

## Methods

**Figure 2:**
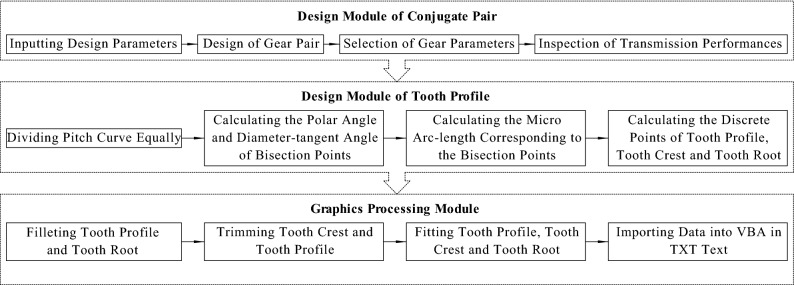
Structure diagram of the CAD system.

There are mainly two kinds of development methods^21^ for a non-circular gear computer-aided design (CAD) system, namely, the generating envelope method and the analytical method. The design accuracy of the generating envelope method depends on the increment of generating motion. However, it has certain errors and application limitations compared with the theoretical tooth profiles. Meanwhile, the analytical method can design accurate tooth profiles according to the mathematical model. Hence, this article adopts the mixed programming of MATLAB and VBA based on this method^17^. As shown in Fig. 2, the CAD system developed consists of three modules: the Design Module of Conjugate Pair, Design Module of Tooth Profile, and Graphics Processing Module. The first two modules obtain the discrete points of the tooth profile, tooth root, and tooth crest of the driving and driven gears using MATLAB to calculate and output them in text. The last module reads the above text data in VBA, controls AutoCAD objects to implement such tasks as automatic fitting, filleting, and trimming, and then finally realizes accurate drawing.

### Dividing pitch curve equally

**Figure 3:**
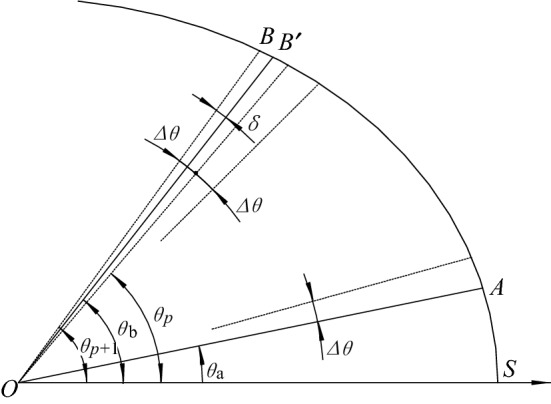
Structure diagram of dividing pitch curve with equal spaces.

The normal tooth thickness on the pitch curve is equal to the tooth space width^2^. To design the gear tooth profile, the pitch curve is divided into 2 $z$ segments. As shown in Fig. 3, assuming that point A is the upper bisection point, the next bisection point $B$ is obtained using the equal polar angle subdivision method, with the following steps:

- Step 1: The polar angle of the pitch curve of the piecewise deformed elliptical gear increases progressively as $\Delta \theta$. To ensure the calculating precision, $\Delta \theta$ should be two orders of magnitude smaller than $\mathrm{\angle }AOB$.
- Step 2: Take ${\theta }_{a}$+$\Delta \theta$ as the polar angle and calculate the arc length ${l}_{j}$ according to Eq. (36).
- Step 3: If ${l}_{j}-\stackrel{\frown}{SA}\le {s}_{n}$, another $\Delta \theta$ is added to the polar angle. Take ${\theta }_{a}$+$2\Delta \theta$ as the new polar angle and repeat the calculation of ${l}_{j}$, and so on. Until ${\theta }_{p+1}$=${\theta }_{a}$+$\left(p+1\right)\Delta \theta$, ${l}_{j}-\stackrel{\frown}{SA}>{s}_{n}$. Thus, ${\theta }_{p}$= ${\theta }_{a}$+$p\Delta \theta$ is taken as the polar angle of the pitch curve of the next bisection point B.
- Step 4: According to the polar angle ${\theta }_{p}$, the coordinates of the bisection point $B$ can be obtained according to Eq. (2).

As shown in Fig. 3, theoretically, there is some error in the bisection point $B$ obtained using the equal polar angle subdivision method. If ${B}^{{\prime}}$ is the next bisection point, in theory, the error of the polar angle is $\delta$ on bisection point $B$. As long as $\Delta \theta$ is small enough, $\delta$ can be controlled to meet $\delta \le \Delta \theta$, which is an acceptable accuracy in engineering.

36 $${l}_{j}={\sum }_{k=1}^{j-1}{\int }_{{\sum }_{k=1}^{j-2}\frac{2\pi }{N{m}_{k}}}^{{\sum }_{k=1}^{j-1}\frac{2\pi }{N{m}_{k}}}\sqrt{{r}_{k-1}^{2}+{\left(\frac{\mathrm{d}{r}_{k-1}}{\mathrm{d}\theta }\right)}^{2}}\mathrm{d}\theta +{\int }_{{\sum }_{k=1}^{j-1}\frac{2\pi }{N{m}_{k}}}^{{\theta }_{\mathrm{p}}}\sqrt{{r}_{k}^{2}+{\left(\frac{\mathrm{d}{r}_{k}}{\mathrm{d}\theta }\right)}^{2}}\mathrm{d}\theta $$

### Curves of tooth crest and tooth root

**Figure 4:**
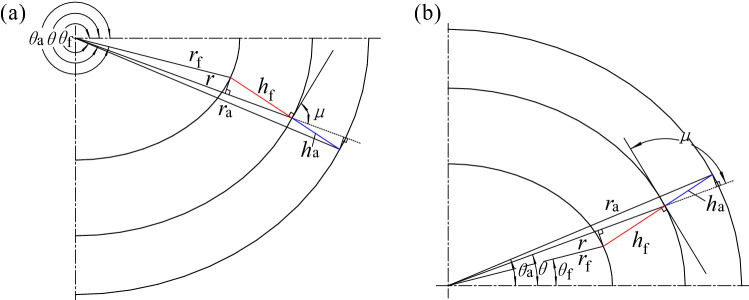
Tooth crest curve and tooth root curve. (**a**) $\upmu \in [0,\uppi /2)$, and (**b**) $\upmu \in [\uppi /2,\uppi )$.

The mathematical model of the tooth profile of non-circular gears^2^ can be adopted in this article. However, the existing literature regards the tooth crest curve and tooth root curve as non-circular curves similar to the pitch curve^22^, which will cause non-negligible design error of tooth height and contact ratio error accordingly, leading to the interference of conjugate pair to be unable to drive. As shown in Fig. 4, for external meshing gears, the tooth crest curve and tooth root curve are theoretically the normal isometric lines of the pitch curve, and their normal distances from the pitch curve are the addendum ${h}_{a}$ and dedendum ${h}_{f}$, respectively.

As shown in Fig. 4a, while $\mu \in$[0, $\pi /2)$, see Eq. (37) for the equation of the tooth crest curve and Eq. (38) for that of the tooth root curve.

37 $$\left\{\begin{array}{l}{r}_{a}=\sqrt{{\left[{h}_{a}\,\mathrm{sin}\,\left(\pi /2-\mu \right)\right]}^{2}+{\left[{r+h}_{a}\,\mathrm{cos}\,\left(\pi /2-\mu \right)\right]}^{2}}=\sqrt{{h}_{a}^{2}+{r}^{2}+2r{h}_{a}\,\mathrm{sin}\,\mu }\\ {\theta }_{a}=\theta -{\,\mathrm{sin}}^{-1}\left({h}_{a}\,\mathrm{cos}\,\mu /{r}_{a}\right)\quad \mu \in [0,\pi /2)\end{array}\right.$$

38 $$\left\{\begin{array}{l}{r}_{f}=\sqrt{{\left[{h}_{f}\,\mathrm{sin}\,\left(\pi /2-\mu \right)\right]}^{2}+{\left[{r+h}_{f}\,\mathrm{cos}\,\left(\pi /2-\mu \right)\right]}^{2}}=\sqrt{{h}_{f}^{2}+{r}^{2}+2r{h}_{f}\,\mathrm{sin}\,\mu }\\ {\theta }_{f}=\theta +{\,\mathrm{sin}}^{-1}\left({h}_{f}\,\mathrm{cos}\,\mu /{r}_{f}\right)\quad \mu \in [\pi /2, \pi )\end{array}\right.$$

As shown in Fig. 4b, while $\mu \in$[$\pi /2$,$\pi )$, see Eq. (39) for the equation of the tooth crest curve and Eq. (40) for that of the tooth root curve.

39 $$\left\{\begin{array}{l}{r}_{a}=\sqrt{{\left[{h}_{a}\,\mathrm{sin}\,\left(\mu -\pi /2\right)\right]}^{2}+{\left[{r+h}_{a}\,\mathrm{cos}\,\left(\mu -\pi /2\right)\right]}^{2}}=\sqrt{{h}_{a}^{2}+{r}^{2}+2r{h}_{a}\,\mathrm{sin}\,\mu }\\ {\theta }_{a}=\theta +{\,\mathrm{sin}}^{-1}\left({h}_{a}\,\mathrm{cos}\,\mu /{r}_{a}\right)\quad \mu \in [0,\pi /2)\end{array}\right.$$

40 $$\left\{\begin{array}{l}{r}_{f}=\sqrt{{\left[{h}_{f}\,\mathrm{sin}\,\left(\mu -\pi /2\right)\right]}^{2}+{\left[{r+h}_{f}\,\mathrm{cos}\,\left(\mu -\pi /2\right)\right]}^{2}}=\sqrt{{h}_{f}^{2}+{r}^{2}+2r{h}_{f}\,\mathrm{sin}\,\mu }\\ {\theta }_{f}=\theta -{\,\mathrm{sin}}^{-1}\left({h}_{f}\,\mathrm{cos}\,\mu /{r}_{f}\right)\quad \mu \in [\pi /2, \pi )\end{array}\right.$$

### Automatic trimming and filleting

The curve of the tooth profile calculated using the analytical method usually exceeds that of the tooth crest, and the part outside the tooth crest needs to be trimmed. There is no function to deal with Trim in VBA, so it cannot be programmed directly. However, the SendCommand^23^ in VBA can accept the same statement as inputting in the Command Line; that is, it can accept AutoLISP functions and point coordinates in the same format and directly operate on curve objects. Table 1^23^ presents the functions used to convert a VBA form to a LISP form. The programming ideas of subroutines of automatic trimming and filleting are as follows:

**Table 1:** Conversion function in VBA.

Functions	Meaning
axPoint2lspPoint	Convert the point format from VBA to SendCommand
axEnt2lspEnt	Convert the metafile format from VBA to SendCommand
GetDoubleEntTable	Convert the metafile and point in VBA to the binary table in SendCommand

- Sub Trim (): Subroutine of automatic trimming. Call the Trim command by using SendCommand. Take the tooth crest curve as the scissor. Call the binary table function to pick up the cutting object. Finally, cut the left and right tooth profile curves of each tooth.
- Sub Break (): Subroutine of automatic breaking. Call the Break command by using SendCommand to break the tooth crest curve at each tooth space position. Call the Trim command. Take the left and right tooth profile curves of each tooth as scissors to cut the tooth crest curve. Finally, form each tooth crest.
- Sub Fillet (): Subroutine of automatic filleting. Call the Break command to break the tooth root curve at the position of each tooth profile. Call the Fillet command through SendCommand for combination. Finally, call the Handle data of each tooth profile and tooth root curve segment to realize automatic filleting.

### Case

**Figure 5:**
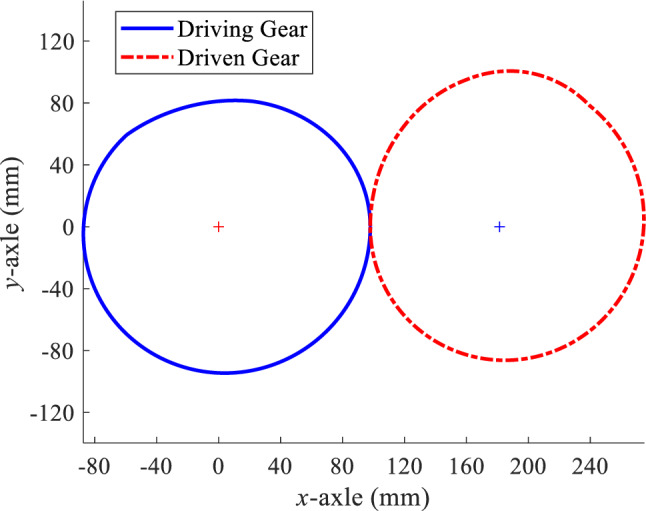
Pitch curves of the conjugate pair.

The design requirements of an external meshing three-segment deformed elliptical gear pair in a variable-speed drive mechanism are as follows: $e$= 0.1, ${m}_{1}$= 1.6, and ${m}_{2}$= 2.0, with an order ${m}_{n}$= 4 mm and $\beta$= 13.872°. The driving gear rotates at a uniform angular velocity, *ω* = 0.628 rad/s. Using the non-circular gear CAD system developed in this article, the main parameters of the conjugate pair are obtained as follows: $A$ = 89.030 mm, $a$= 181.462 mm, ${m}_{3}$= 0.533, $z$= 44, and ${z}^{{\prime}}$= 44. Figure 5 shows the pitch curves of the designed external meshing three-segment deformed elliptical gear pair. The left side (blue solid line) is the pitch curve of the three-segment deformed elliptical gear (driving gear), while the right side (red dash-dotted line) is the pitch curve of the driven gear, also consisting of three segments.

**Figure 6:**
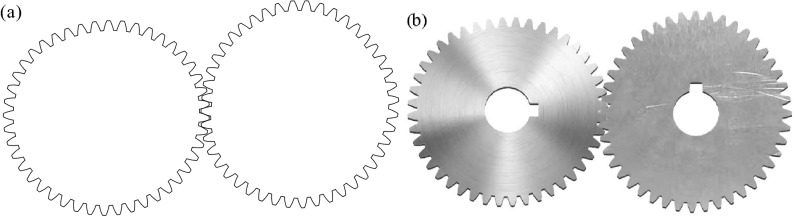
Three-segment deformed elliptical gear pair. (**a**) Transverse section. (**b**) Gear pair manufactured through wire-electrode cutting, and the background of them is deducted using Adobe Photoshop CS5.

Figure 6a shows the transverse section of the conjugate pair designed by the CAD system developed in this article. Figure 6b shows the actual gear pair manufactured through wire-electrode cutting. The experiment shows that it can mesh and drive correctly.

**Figure 7:**
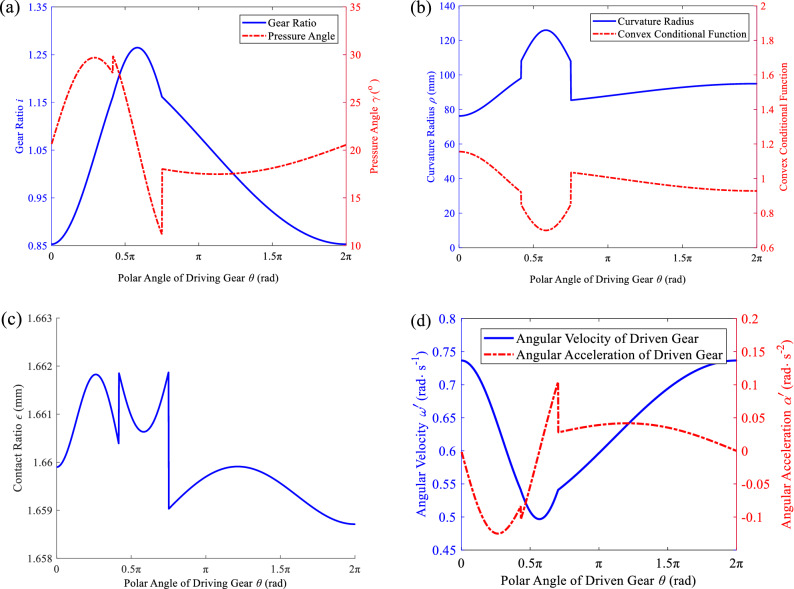
Transmission performances of the conjugate pair. (**a**) Gear ratio and pressure angle. (**b**) Curvature radius of the driving and driven gears. (**c**) Contact ratio. (**d**) Angular velocity and angular acceleration of the driven gear.

As shown in Fig. 7a, the gear ratio curve (blue solid line) is divided into three segments, which correspond to the pitch curve of the driving gear and $\theta$, and the two adjacent segments are continuous at the connection. From the pressure angle curve (red dash-dotted line), $\upgamma$ ∈ [11.184°, 29.867°], and then $\mu \in$ [81.184°, 99.867°], which meets the transmission requirements. As shown in Fig. 7 (b), the blue solid line is the curvature radius of the pitch curve of the driving gear at each θ. As presented in Table 2, the maximum eccentricity of the non-concave pitch curve for segments 1–3 is ${{e}_{1}}_{\mathrm{max}}$= 1.000, ${{e}_{2}}_{\mathrm{max}}$= 0.334, and ${{e}_{3}}_{\mathrm{max}}$= 1.000, respectively, which are greater than the design parameter $e$ (0.1). In addition, as presented in Table 2, ${\rho }_{\mathrm{min}}$= 76.246 mm from Table 2, so the pitch curve of the driving gear is fully convex. As shown in Fig. 7b, the red dash-dotted line is the curvature radius of the pitch curve of the driven gear at each $\theta$. As presented in Table 2, ${\rho }_{\mathrm{min}}^{{\prime}}$= 69.413 mm, so it can also be verified that the pitch curve of the driven gear is also fully convex. Therefore, the driving and driven gears can be processed by the hobbing or shaping^3,17^. As presented in Table 2, the maximum normal modulus of the driving and driven gears without undercutting in the hobbing process is ${{m}_{\mathrm{n}}}_{\mathrm{max}}$= 9.396 mm and ${{m}_{\mathrm{n}}^{{\prime}}}_{\mathrm{max}}$ = 0.8.554 mm, respectively, both of which are greater than ${m}_{\mathrm{n}}$ = 4 mm. Hence, hobbing can be used. The minimum teeth number of the driving and driven gears without undercutting in the shaping processing is ${{z}_{0}}_{\mathrm{min}}$=  − 31 and ${{z}_{0}^{{\prime}}}_{\mathrm{min}}$=  − 30, respectively. Since the actual teeth number of gear-shaping cutter is greater than zero, it can be seen that the shaping process can be adopted.

**Table 2:** Parameters for inspection.

Parameters	${{\mathrm{e}}_{1}}_{\mathrm{max}}$	${{\mathrm{e}}_{2}}_{\mathrm{max}}$	${{\mathrm{e}}_{3}}_{\mathrm{max}}$	${\uprho }_{\mathrm{min}}$(mm)	${\uprho }_{\mathrm{min}}^{\mathrm{^{\prime}}}$(mm)	${{\mathrm{m}}_{\mathrm{n}}}_{\mathrm{max}}$(mm)	${{\mathrm{m}}_{\mathrm{n}}^{\mathrm{^{\prime}}}}_{\mathrm{max}}$(mm)	${{\mathrm{z}}_{0}}_{\mathrm{min}}$	${{\mathrm{z}}_{0}^{\mathrm{^{\prime}}}}_{\mathrm{min}}$
Calculated value	1	0.334	1	76.246	69.413	9.396	8.554	− 31	− 30
Allowable limits	Convex condition: $\ge \mathrm{ e}$(0.1)			Convex condition: $\uprho \ge$ 0		Non-undercutting condition by hobbing: $\ge 4$		Always satisfied	

As shown in Fig. 7c, the contact ratio $\varepsilon$ ∈ [1.659, 1.662] is greater than 1, which meets the continuous transmission conditions. As shown in Fig. 7d, the angular velocity curve (blue solid line) of the driven gear is composed of three continuous curves. Moreover, there is an abrupt change in the angular acceleration curve (red dash-dotted line) of the driven gear at the pitch curve section. Therefore, there is a flexible impact but no rigid impact during transmission^24^, which is not suitable for high-speed transmission.

## Conclusions

The pitch curve of a piecewise deformed elliptical gear is an end-to-end closed continuous curve. The shape of the pitch curve of a non-circular gear can be adjusted by adjusting $A$, $e$, $N$, and ${m}_{k}$. Compared to that of the deformed elliptical gear, the shape of the pitch curve of the piecewise deformed elliptical gear is more changeable, giving more convenience in adjusting the gear ratio and expanding the application field of non-circular gears. Piecewise deformed elliptical gears unify all kinds of elliptical gears, and the latter is a special case of the former. When $N$= 1 and ${m}_{1}$ is a positive integer, the piecewise deformed elliptical gear is transformed into a high-order elliptical gear, and the driven gear meshed with it is also a high-order elliptical gear. When $N$= 1 and ${m}_{1}$= 1, it further evolves into an elliptical gear pair. When $N$= 2, the piecewise deformed elliptical gear is transformed into a deformed elliptical gear, and the driven gear meshed with it is also a deformed elliptical gear. When $N$= 2 and ${m}_{1}$= ${m}_{2}$= 1, it also further evolves into an elliptical gear. When $N\ge$ 3, the driven gear is a non-circular gear with a free pitch curve and is no longer a piecewise deformed elliptical gear. In addition, for the piecewise deformed elliptical gear pair, the $N$ of the driving gear is equal to the ${N}^{{\prime}}$ of the driven gear, which can only be used for external meshing transmission.

This paper constructs the conjugate pair design method and performance inspection method of piecewise deformed elliptical gear and provides the CAD system development technology. The design case verifies the above theory. The designed conjugate pair can realize correct meshing and can be applied to practical transmission. For the piecewise deformed elliptical gear pair with $N\ge$ 3, there is flexible impact but no rigid impact in the transmission process, so it is not suitable for high-speed transmission. However, when $N\le$ 2, it is transformed into elliptical gear transmission or deformed elliptical gear transmission without the above disadvantages.

## Supplementary Information

Supplementary Information.

## Data Availability

The raw data analyzed/used during the study is included in supplementary file.

## List of symbols

$a$: Center distance (mm)
$$e$$: Eccentricity,$$e$$ ∈ [0, 1]
$${h}_{a}^{*}$$: Addendum coefficient
$${h}_{a}$$: Addendum (mm)
$$i$$: Gear ratio
$$j$$, $$k$$: Tally marks
$$m$$: Modified coefficient
$$\mathrm{max}$$: Maximum
$$\mathrm{min}$$: Minimum
${m}_{n}$: Normal modulus (mm)
${m}_{t}$: Transverse module (mm)
$$n$$: Order
$r$: Polar radius of the driving gear (mm)
${r}^{{\prime}}$: Polar radius of the driven gear (mm)
$$z$$: Teeth number
${z}_{0}$: Teeth number of shaper
$A$: Semimajor axis (mm)
$L$: Pitch curve perimeter (mm)
$N$: Segment number of the driving gear
${N}^{{\prime}}$: Segment number of the driven gear
$\alpha$: Angular acceleration of the driving gear (rad·s^−2^)
${\alpha }^{{\prime}}$: Angular acceleration of the driven gear (rad·s^−2^)
${\alpha }_{n}$: Tooth profile angle in a normal plane (rad)
${\alpha }_{t}$: Tooth profile angle in a transverse plane (rad)
$\beta$: Helix angle (rad)
$\theta$: Polar angle of the driving gear (rad)
${\theta }^{{\prime}}$: Polar angle of the driven gear (rad)
$\rho$: Curvature radius of the driving gear (mm)
${\rho }^{{\prime}}$: Curvature radius of the driven gear (mm)
$$\delta $$: Allowable error
$\gamma$: Pressure angle (rad)
$\mu$: Diameter-tangent angle (rad)
$$\varepsilon $$: Contact ratio, $$\varepsilon $$ ≥ 1
*ω*: Angular velocity (rad·s^−1^)
